# A New Integrated Lab-on-a-Chip System for Fast Dynamic Study of Mammalian Cells under Physiological Conditions in Bioreactor

**DOI:** 10.3390/cells2020349

**Published:** 2013-05-27

**Authors:** Janina Bahnemann, Negar Rajabi, Grischa Fuge, Oscar Platas Barradas, Jörg Müller, Ralf Pörtner, An-Ping Zeng

**Affiliations:** 1 Institute of Bioprocess and Biosystems Engineering, Hamburg University of Technology, Denickestraße 15, 21073 Hamburg, Germany; E-Mails: janina.bahnemann@tuhh.de (J.B.); grischa.fuge@tuhh.de (G.F.); o_platas@tuhh.de (O.P.B.); poertner@tuhh.de (R.P.); 2 Institute of Microsystems Technology, Hamburg University of Technology, Eissendorfer Strasse 42, 21073 Hamburg, Germany; E-Mails: negar.rajabi@tuhh.de (N.R.); mueller@tuhh.de (J.M.)

**Keywords:** biomicrofluidics, lab-on-a-Chip, mammalian cells, metabolism, integrated bioreactor

## Abstract

For the quantitative analysis of cellular metabolism and its dynamics it is essential to achieve rapid sampling, fast quenching of metabolism and the removal of extracellular metabolites. Common manual sample preparation methods and protocols for cells are time-consuming and often lead to the loss of physiological conditions. In this work, we present a microchip-bioreactor setup which provides an integrated and rapid sample preparation of mammalian cells. The lab-on-a-chip system consists of five connected units that allow sample treatment, mixing and incubation of the cells, followed by cell separation and simultaneous exchange of media within seconds. This microsystem is directly integrated into a bioreactor for mammalian cell cultivation. By applying overpressure (2 bar) onto the bioreactor, this setup allows pulsation free, defined, fast, and continuous sampling. Experiments evince that Chinese Hamster Ovary cells (CHO-K1) can be separated from the culture broth and transferred into a new medium efficiently. Furthermore, this setup permits the treatment of cells for a defined time (9 s or 18 s) which can be utilized for pulse experiments, quenching of cell metabolism, and/or another defined chemical treatment. Proof of concept experiments were performed using glutamine containing medium for pulse experiments. Continuous sampling of cells showed a high reproducibility over a period of 18 h.

## 1. Introduction

Fast and dynamic experiments with mammalian cells under real physiological conditions are essential for quantitative and systematical studies of cellular metabolism and its regulation. In particular, the quantitative determination of intracellular metabolites and metabolic fluxes requires rapid and specific sample preparation under physiological conditions [1,2]. Furthermore, regarding the compartmentalization of metabolic pathways in mammalian cells, a separated analysis of mitochondrial and cytosolic metabolites is essential to expand our knowledge of the *in vivo* dynamics of key metabolic reactions [3,4]. Conventional approaches using standard manual lab procedures cannot fulfill these requirements. This is due to the fact that the time (in the range min-hour) required for manual handling exceeds the time scale (ms-s) of biological reactions by orders of magnitude. Integrated microfluidic systems offer a promising tool to circumvent these time limitations and provide additional functionalities based upon altered relations between physical forces on the microscale. Cell and particle separation employing microfluidics recently gained significant attention in sample preparation for biological and chemical studies [5,6]. Microfluidic systems provide much lower sample and reagent consumption, a large surface to volume ratio, rapid and precise sample treatment, as well as a high automation potential compared to common macroscale devices [7,8,9]. Furthermore, microfluidic systems can be used to generate controlled environments in which metabolomics and cellomics experiments can be conducted under defined conditions in a reproducible manner [10]. Their key feature is the capability to assemble the necessary components to answer a specific question in one device. For instance, drug metabolism has been imitated by a combination of bioreactor, cytotoxicity and solid phase extraction modules [11]. Also qualitative and quantitative metabolism studies with mammalian cells were conducted by direct integration of electrospray ionization mass spectrometry in a lab-on-a-chip setup [12].

In previous experiments, the application of mammalian cells by selective permeabilization on a chip has been successfully demonstrated in order to enable discrete metabolite measurements [13]. The presented lab-on-a-chip (LoaC) integrates the functions of rapid mixing, defined incubation times and the separation of subcellular components. Furthermore, it offers the possibility of a controlled cell lysis [14] and the performance of substrate pulse experiments. In the present study, the microchip has been directly connected to a bioreactor for mammalian cell cultivation. This microchip-bioreactor setup provides continuous sampling of mammalian suspension cells and the direct sample preparation on chip. Due to a combination of two mixing and two incubation modules as well as a cell separation unit at the end, the LoaC allows various biological and chemical applications for the treatment of cells. In this proof-of-concept study we apply the integrated LoaC for dynamic pulse experiments in order to investigate the impact of different medium conditions on the metabolic state of mammalian cells.

## 2. Experimental Section

### 2.1. Mammalian Cell Cultivation

The CHO-K1 cell line was obtained from the University Bielefeld (AG Noll). The cells were cultured in suspension in defined serum- and protein-free TC-42 medium (TeutoCell, Bielefeld, Germany) supplemented with 4 mM L-glutamine (PAA). This culture medium contained a standard concentration of 40 mM glucose. Precultures of CHO-K1 were grown in 250 mL Erlenmeyer flasks with baffles and air filter (Corning Inc.) with a working volume of 100 mL. The cultivation vessels were incubated on a shaking device (225 rpm) at 37 °C in a humid atmosphere supplemented with 5% CO_2_. 

The main experiments with CHO-K1 culture were performed in a VSF2000 bioreactor (Bioengineering, Switzerland) with a starting culture volume of 1.5 L. The bioreactor was inoculated with a density of 2 × 10^6^ cells/mL using precultured cells which were harvested during the exponential growth phase with a viability of ≥ 98%. The cultivation temperature was set to 37 °C and the impeller (Rushton 6-blade) speed was set to 300 rpm. During the cultivation, the pH value was controlled at 7.2 using 0.5 M sodium carbonate solution. The gas flow was connected to the top of the bioreactor and individually regulated to create an overpressure between 1 and 2.5 bar.

### 2.2. Design of the Microfluidic System

**Figure 1:**
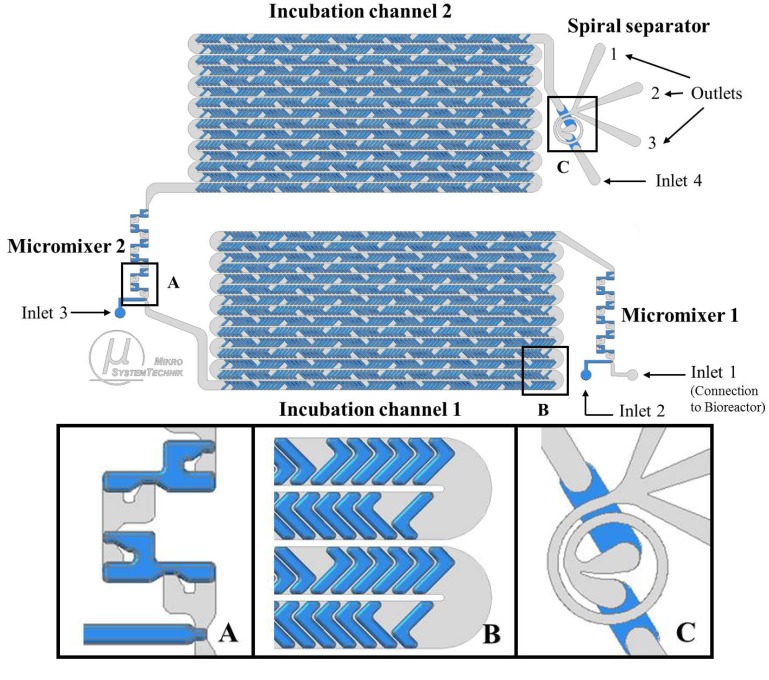
Design of the integrated continuous flow LoaC: Chip modules, pulse addition, mixing, incubation and further cell separation. The detailed view on the chip modules is depicted below: Micromixer **(A)**, incubation channel **(B)**, and spiral separator **(C)**. Structures etched into silicon are shown in grey and structures etched into glass are shown in blue.

The first step of our proof-of-concept study was the design and fabrication of the microfluidic system. Our LoaC design consists of five connected microfluidic modules integrated on a single chip as shown in Figure 1.

The first module is a split-and-recombine (SAR) micromixer (Micromixer 1) for rapid mixing of cell suspension [15] with L-glutamine containing medium. This module contains two inlets. Inlet 1 is directly connected to the bioreactor for continuous cell harvesting. Inlet 2 is attached to a syringe pump. This inlet can be used to mix a defined substrate pulse or a solution of choice in milliseconds with the cell sample. In the three dimensional channel geometry fluids are split in one mixing unit and recombined again in the next unit. This mechanism doubles the number of fluid layers after each mixing unit, resulting in a stack of thin fluid layers with low diffusion lengths [15]. Furthermore, mixing is enhanced by chaotic advection in the 90° turns of channel path, which plays a beneficial role in the fluid layering in the recombination regions [15].

The second stage composes the incubation channel 1 that consists of one meander-shaped channel. The channel design is based on staggered herringbone mixer (SHM) structures and provides a residence time of about 9 sec. The fluid flow is stirred by vortices caused by the SHM grooves [16], such that the overall residence time distribution of cells is lowered significantly.

This incubation unit is followed by Micromixer 2, which is a similar design to the first micromixer of the system. This mixer has a further inlet (Inlet 3, attached to a syringe pump) which is used to insert another substrate pulse. This additional inlet can also be used for the addition of a quenching solution or chemical reagents, which was already demonstrated in previous experiments [13]. The second mixing module is connected with a second incubation channel, which has the same design as the first incubation channel. A spiral separator forms the last unit of the LoaC, before the cells exit the chip for off-chip analysis. It consists of three outlets and one inlet (Inlet 4, attached to a syringe pump) that can be used to add a wash medium (e.g., physiological salt solution (0.9% NaCl) or phosphate buffered saline (PBS)). The spiral separator ensures the separation of cells and extracellular medium at a defined flow rate. At the same time, the initial carrier medium of the cells is exchanged rapidly by the wash medium in a continuous flow [14]. The underlying mechanism of the spiral separator is inertial focusing [14,17]. Cells are injected at the inner wall, when coming from the previous microfluidic module, while the wash medium is injected at the outer wall of the curved channel in the spiral separator. When the cells flow through the curved channel, they move away from their initial position and find an equilibrium position at the outer wall in the cross-section of the curved channel, where the Dean force is balanced by lift forces (causing inertial migration) [14,17]. However, the majority of the initial medium is still located close to the inner wall. Thus the cells are transferred into the new medium at their new equilibrium position caused by inertial focusing. 

### 2.3. LoaC Fabrication

The LoaC is fabricated in bonded glass-silicon-glass substrates by conventional microfabrication techniques using photolithography. Channels in the silicon substrate were structured in a deep reactive ion etching (DRIE) process. In order to structure channels in the glass substrate, 49% hydrofluoric acid (HF) was used in a wet chemical etching process. The substrates were bonded anodically to ensure an irreversible and leakage-free connection. The finalized chip is heated homogenously to 37 °C at the two incubation channels by attached heating foils (Kapton, Telemeter Electronic GmbH, Germany). Thus, physiological conditions on the chip are provided during the experiments.

### 2.4. Integrated Microchip-Bioreactor: Experimental Setup

**Figure 2:**
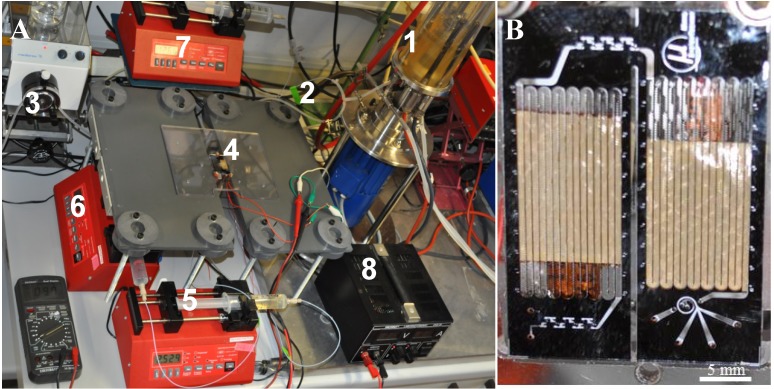
(**A**) Experimental setup: Integrated system of microchip and bioreactor. 1: VSF2000 Bioreactor, 2: Wash loop for microchip and bioreactor connection, 3: Buffer pump, 4: Integrated microchip system 5: Syringe pump for substrate pulse or reagent (Inlet 2), 6: Syringe pump for a second substrate pulse or reagent (Inlet 3), 7: Syringe pump for washing buffer, 8: Power supply; (**B**) Detailed view on the fabricated integrated microchip.

Microfluidic experiments were carried out subsequently after connecting the bioreactor to the microsystem (Inlet 1). During the experiments, the bioreactor was surrounded by a steel jacket allowing the application of overpressure up to 2.5 bar. Syringe pumps (AL1000, World Precision Instruments Germany GmbH) were assembled with common luer-lock syringes, filled with sterile filtrated media, buffers or other solutions required. The syringes were connected to the microchip inlets 2, 3 and 4 (see Figure 1, Figure 2) using capillary tubes. The experimental setup of the integrated microchip and bioreactor is shown in Figure 2A. 

Pump flow rates were determined in previous experiments [11] and set to constant values:

Inlet 2 (pulse medium or reagent): 250 µL/min
Inlet 3 (pulse medium or reagent): 250 µL/min
Inlet 4 (wash or quenching medium): 1450 µL/min

The flow rate and the separation efficiency at different pressures were estimated in advance to provide optimal experimental conditions. According to the results (see 3.1) further microfluidic experiments were performed by applying 2 bar (flow rate of 300 µL/min) onto the bioreactor. The total flow rate on the chip was 2.25 mL/min.

Each experiment was carried out continuously for a period of 45 min. Samples were collected at the outlets of the chip in individual sterile tubes. Samples that were prepared for metabolic analysis were directly collected on dry ice in order to reduce metabolic activity. The integrated LoaC was driven for more than 18 h in total with washing steps (0.9% NaCl solution) after each experiment. The fabricated LoaC is presented in Figure 2B.

### 2.5. Glutamine Pulse Experiments

To perform metabolic pulse experiments on the chip, CHO-K1 cells were first cultured under glutamine starvation conditions. For this purpose, the bioreactor was inoculated as described in 2.1, except that TC-42 medium without L-glutamine was used. The cells were cultured under glutamine-free conditions for 6 h before starting the pulse experiments. Glutamine pulse experiments were carried out with TC-42 medium supplemented with 4 mM *L*-glutamine (PAA, Germany). The L-glutamine containing medium was tempered to 37 °C, filled into sterile syringes, and injected individually by syringe pumps connected to Inlet 2 and Inlet 3 at a flow rate of 250 µL/min. For a pulse of 9 s, the syringe pump connected to Inlet 3 was operated, while the pump at Inlet 2 was turned off. To have a pulse of 18 s, the syringe pump connected to Inlet 2 was operated, while the pump connected to Inlet 3 was turned off. A solution of 0.9% NaCl was continuously inserted at Inlet 4 at a flow rate of 1450 µL/min in order to wash the cells and separate them from their culture medium. As a reference, CHO-K1 cells from bioreactor were conveyed through the LoaC without exposing a glutamine pulse (0 s). Samples exiting the chip outlets were directly collected in individual tubes on dry ice.

### 2.6. Cell Density and Viability

The cell density and viability of CHO-K1 cells before and after the microfluidic experiments were estimated by cell counting using Trypan blue stain.

### 2.7. Analysis of Glucose Concentration

The glucose concentration of collected samples was investigated enzymatically using a multiparameter bioanalytical system (YSI 7100 MBS, YSI Incorporated Life Sciences, USA). The culture medium (TC-42, see 2.1) used for the experiments contained a standard concentration of 40 mM glucose. The culture medium distribution in the samples was determined as a measure of the remaining glucose concentration.

### 2.8. Fluorescence Staining of CHO-K1 Cells

For visualization of the cell flow in the microchip, CHO-K1 cells were stained with Calcein AM (Sigma Aldrich, USA) prior to use. Calcein AM results in a green fluorescent color of mammalian cells. 2 × 10^6^ cells/mL were incubated at 37 °C for 10 min in a 2µM Calcein AM working solution. Stained cells were injected by a syringe pump connected to the microchip Inlet 1 at a flow rate of 300 µL/min. Visualization of green fluorescent cells was performed under fluorescence microscope (C1, Nikon Corp.) and pictures were taken with a CCD camera (DS-U1, Nikon Corp.) attached to the microscope. 

## 3. Results and Discussion

### 3.1. Medium Exchange and Separation of CHO-K1 Cells

**Figure 3:**
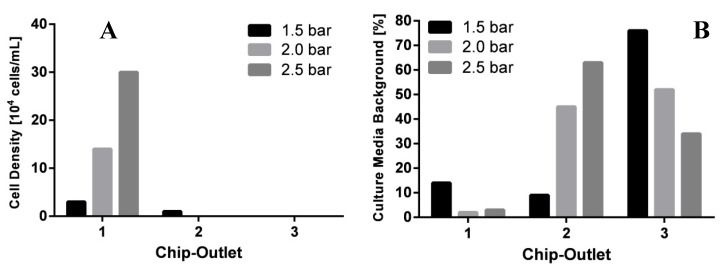
(**A**): Effects of overpressure on the distribution of Chinese Hamster Ovary cells (CHO-K1) cells at the chip outlets 1, 2 and 3 after passing the spiral separator. (**B**): Distribution of extracellular medium (as measure of glucose concentration) depending on overpressure at chip Outlets 1, 2 and 3 after passing the spiral separator.

To provide continuous experiments on chip, the LoaC was directly connected to a bioreactor for mammalian cell culture. The separation efficiency of CHO-K1 cells was tested under different flow rates on chip by applying overpressure on the bioreactor setup (described in 2.4). By the applied pressures, the CHO-K1 cell suspension was successfully injected from the bioreactor into the microsystem in a pressure-driven flow. Samples were collected at the three outlets of the spiral separator. The viability of collected cells was ≥ 98% in all cases. The separation efficiency of culture medium and CHO cells dependent on the pressure is presented in Figure 3A,B.

As shown in Figure 3A, the density of cells collected at Outlet 1 increased due to an increase of pressure. At a pressure of 1.5 bar, a low cell density of 3 × 10^4^ cells/mL was detected at Outlet 1, while some cells also passed through Outlet 2. In contrast, 100% of inserted CHO-K1 cells accumulated at Outlet 1 at pressures of 2 and 2.5 bar. An average cell density of 15 × 10^4^ cells/mL was obtained at a pressure of 2 bar while even 30 × 10^4^ cells/mL were collected at a pressure of 2.5 bar. For further metabolic analysis, a high total amount of cells is required to circumvent analytical detection limits. Thus, high cell densities in samples are preferred. At the same time, efficient separation of the cells from their culture medium is required. Hence, low glucose concentrations at Outlet 1 are desired. The glucose distribution in the chip Outlets 1, 2 and 3 dependent on the pressure is shown in Figure 3B. At 1.5 bar, relatively high glucose concentrations (14%) were determined in outlet 1. The use of 2 bar resulted in the lowest glucose concentrations at Outlet 1 (2%). Further increase of pressure to 2.5 bar caused an increase of glucose concentration in Outlet 1 (3%). Therefore, 2 bar were chosen as operating conditions since it allows the most efficient removal of glucose from the cells, although higher pressure would yield more cells at the same experimental time.

These results demonstrate that a representative amount of CHO-K1 cells was efficiently transferred from their culture medium to the washing solution at 2 bar. In this experiment, all syringes were filled with 0.9% NaCl solution. This physiological solution served as testing solution to keep the flow rates within the microsystem constant without harming the living cells. For further biological experiments, any other solution or reagent with a similar viscosity has the potential to be used instead. It should be mentioned that the set-up of the bioreactor with overpressure presents certain requirements (e.g., hardware demand, setup time and high media consumption) but provide well defined culture conditions. In some cases it may be desirable to integrate the microfluidic chip in a less complex setup. By design, the chip is also suitable to work with other devices that work in a similar pressure range, *i.e.* syringe pumps. However, limitations inherent to those devices (lower amount of cells, limited control over culture conditions regarding pH, temperature and sedimentation) apply and have to be accounted for. Alternatively, smaller and/or simpler bioreactors can be used.

**Figure 4:**
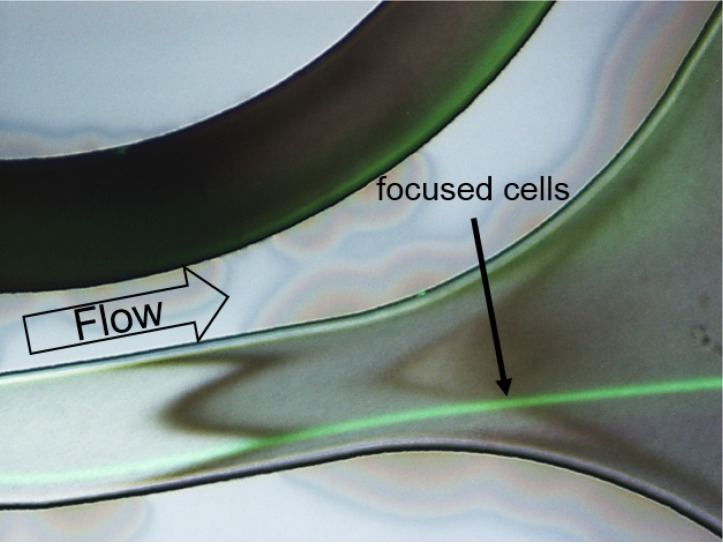
Overlay of a fluorescence reflected-light microscopy image of calcein AM stained CHO-K1 cells (green) crossing the spiral separator at a flow rate of 2.5 mL/min. Detailed view on a cut-out of the fabricated spiral separator close to the outlets. The cells are focused on a single streamline.

For visualization of the flow in the spiral separator, CHO-K1 cells were stained with green fluorescent dye. For better path visualization, an overlay of fluorescence and reflected-light microscopy images is shown. The resulting image is depicted in Figure 4. The CHO-K1 cells, that entered the spiral separator at the inner wall, changed the path from the inner wall to an equilibrium position closer to the outer channel wall at a flow rate of 2.5 mL/min. This flow rate corresponds to a pressure of 2 bar on the bioreactor at the pump rates used in the experiment. The cells are focused in a single stream close to the outer wall during their pass through the spiral separator before exiting the chip. The underlying effect is inertial focusing of particles in the fluid flow, as a result of the superposition of inertial migration and the Dean effect in channels with curvature [17].

### 3.2. Biological Application: Dynamic Pulse experiments

**Figure 5:**
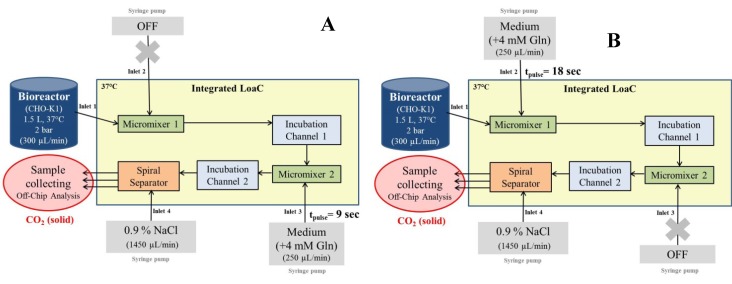
Schematic of the experimental setup for glutamine pulse experiments. (**A**) Experimental setup for a 9 sec pulse. Here, the syringe pump at Inlet 2 is switched off while the pump at Inlet 3 is operating. (**B**) Experimental setup for an 18 sec pulse. Here, the syringe pump at Inlet 2 is operating while the pump at Inlet 3 is switched off.

l-glutamine pulses were applied to the cell suspension on chip. According to our chip design and the experimental setup, we were able to apply three different pulse times on chip: 0 s, 9 s, and 18 s (see Figure 5). Glutamine pulses were successfully provided after a glutamine starvation in CHO-K1 culture. A total of 10^7^ cells were collected for each experimental state (0 s, 9 s, and 18 s) within 45 min of continuous operation per experiment. By collecting the samples directly in dry-ice-cooled containers, a quenching of metabolic reactions was achieved. 

**Figure 6:**
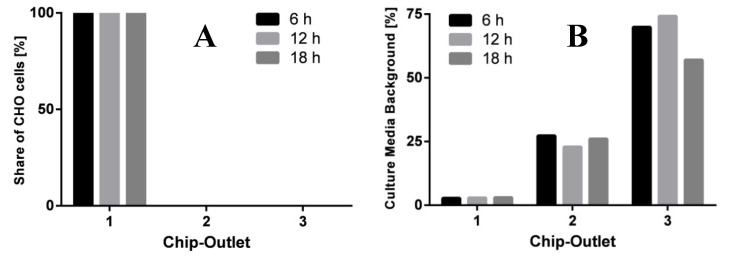
Distribution of CHO-K1 cells (**A**) and extracellular medium (as measure of glucose concentration) (**B**) at chip outlets 1, 2 and 3 after a total operation time of 6 h, 12 h, and 18 h at a pressure of 2 bar.

In this experiment, the microfluidic-bioreactor setup was operated continuously for a period of 18 h in total. As shown in Figure 6, the performance of cell and medium separation did not decrease during the operation. Due to the separation of CHO-K1 cells from the exterior medium, a separate analysis of intracellular and extracellular metabolites can be performed off-chip (work in progress). These results demonstrate that the presented microchip is suitable for dynamic pulse experiments and allows a continuous sample preparation with the integrated bioreactor. In principle, the LoaC setup can be extended by further microfluidic modules and thus would allow additional incubation times and applications.

Since the residence time of the cells in each integrated experimental module is defined by the chip layout and the applied flow rates rather than the experimenter’s accuracy, batch-to-batch variability can be vastly reduced applying this microchip. Additionally, the thin chip geometries allow the utilization of external temperature control. In our case, physiological conditions were chosen but by design the possible temperature range is much wider. In our setup, fixed incubation times of 9 s and 18 s were chosen for the pulse experiments. However, the incubation time on chip can be changed by adjustment of the flow rate (lower or even higher pressure or pump rates). To achieve high cell and media separation at the same time, the pump rate of the syringe pump at inlet 4 (spiral separator) has to be adjusted accordingly. The data published in this article is merely supposed to demonstrate the performance of the described hardware setup. Further experiments focusing on its application and metabolic analysis are on the way. 

Another possible application provided by the presented microchip-bioreactor setup was demonstrated by the selective permeabilization of mammalian cell membranes using digitonin [13,14]. Digitonin is a chemical detergent that has shown the capability to permeabilize plasma membranes at high rate by interacting with the membrane bound cholesterol [18]. Due to the lower cholesterol content in the double lipid layer of cell organelles, a defined digitonin concentration between 0.01–0.02% (w/v) allows a selective permeabilization of plasma membranes, while mitochondrial membranes remain unimpaired [19]. Using this lysis method, intracellular structures as the mitochondria and the cell nucleus are confined in the “ghosts” cells, while the cytoplasm diffuses out of the permeabilized cell membrane after lysis [14]. According to our setup, a combination of a defined substrate pulse and a subsequent permeabilization on chip is conceivable as well. The results demonstrate the high potential of the presented microfluidic-bioreactor setup to provide rapid separation of individual cell compartments and allow further metabolic off-chip analysis [14,15]. 

## 4. Conclusions

The aim of this study was the proof-of-principle application of a microchip-bioreactor setup for dynamic pulse experiments under physiological conditions. The microfluidic chip was connected directly to a bioreactor for automatized cell harvesting at any desired cultivation state. Continuous sampling of cell suspension was achieved by overpressure applied onto the bioreactor. Different flow rates have been tested in order to provide an ideal separation of CHO-K1 cells and culture medium. Best separation of CHO-K1 cells from their native medium was achieved at an overpressure of 2 bar which corresponds to a total flow rate of 2.25 mL/min on the chip. At this flow rate, we collected 15 × 10^4^ cells/mL in average, using a starting cell density of 2 × 10^6^ cells/mL in the bioreactor. The integrated LoaC setup transferred CHO-K1 cells from its surrounding media into metabolite free buffer in a matter of milliseconds without applying as high shear forces as in conventional centrifugation. The viability of collected cells was ≥ 98% in all cases, which demonstrates the gentle preparation process in the microsystem. The overpressure in the bioreactor was also found to not impair the cell viability. In addition to the separation of cells and medium, this microfluidic system also provides defined incubation times of 9 s and 18 s, respectively on a chip. As proof-of-concept, experiments were successfully performed with glutamine containing culture medium. For further biological experiments, any other solution or reagent with a similar viscosity has the potential to be used. Hence, our results indicate that the presented microchip-bioreactor setup enables the performance of continuous biological short-time experiments in microscale. This demonstrates the high potential of the newly developed setup for quantitative and dynamic study of mammalian cells under real cultivation conditions in a bioreactor. 
